# Classification of Cells in CTC-Enriched Samples by Advanced Image Analysis

**DOI:** 10.3390/cancers10100377

**Published:** 2018-10-10

**Authors:** Sanne de Wit, Leonie L. Zeune, T. Jeroen N. Hiltermann, Harry J. M. Groen, Guus van Dalum, Leon W. M. M. Terstappen

**Affiliations:** 1 Department of Medical Cell BioPhysics, University of Twente, 7522 NH Enschede, The Netherlands; s.dewit@utwente.nl (S.W.); l.l.zeune@utwente.nl (L.L.Z.); 2 Department of Applied Mathematics, University of Twente, 7522 NH Enschede, The Netherlands; 3 Department of Pulmonary Diseases, University Medical Center Groningen, University of Groningen, 9713 GZ Groningen, The Netherlands; t.j.n.hiltermann@umcg.nl (T.J.N.H.); h.j.m.groen@umcg.nl (H.J.M.G.); 4 Department of General, Visceral and Pediatric Surgery, University Hospital of the Heinrich-Heine-University Düsseldorf, 40225 Düsseldorf, Germany; Guus.vanDalum@med.uni-duesseldorf.de

**Keywords:** circulating tumor cells, CellSearch^®^, EpCAM, leukocytes, ACCEPT, deep Learning, classification, image analysis, non-small cell lung cancer

## Abstract

In the CellSearch^®^ system, blood is immunomagnetically enriched for epithelial cell adhesion molecule (EpCAM) expression and cells are stained with the nucleic acid dye 4′6-diamidino-2-phenylindole (DAPI), Cytokeratin-PE (CK), and CD45-APC. Only DAPI+/CK+ objects are presented to the operator to identify circulating tumor cells (CTC) and the identity of all other cells and potential undetected CTC remains unrevealed. Here, we used the open source imaging program Automatic CTC Classification, Enumeration and PhenoTyping (ACCEPT) to analyze all DAPI+ nuclei in EpCAM-enriched blood samples obtained from 192 metastatic non-small cell lung cancer (NSCLC) patients and 162 controls. Significantly larger numbers of nuclei were detected in 300 patient samples with an average and standard deviation of 73,570 ± 74,948, as compared to 359 control samples with an average and standard deviation of 4191 ± 4463 (*p* < 0.001). In patients, only 18% ± 21% and in controls 23% ± 15% of the nuclei were identified as leukocytes or CTC. Adding CD16-PerCP for granulocyte staining, the use of an LED as the light source for CD45-APC excitation and plasma membrane staining obtained with wheat germ agglutinin significantly improved the classification of EpCAM-enriched cells, resulting in the identification of 94% ± 5% of the cells. However, especially in patients, the origin of the unidentified cells remains unknown. Further studies are needed to determine if undetected EpCAM+/DAPI+/CK-/CD45- CTC is present among these cells.

## 1. Introduction

Circulating tumor cells (CTC) are cells disseminated from the primary or metastatic tumor site into the bloodstream. In several cancer types, including non-small and small cell lung, prostate, breast, colon, bladder, gastric carcinoma, and melanoma, the presence of CTC in circulation is associated with a poor survival rate of the patients [1,2,3,4,5,6,7,8,9]. With many blood cells circulating in our cardiovascular system, CTC are rare events. A concentration of one CTC per mL of blood is already high, while the concentration of white blood cells is around 5 × 10^6^ cells and red blood cell concentrations reach up to 5 × 10^9^ cells per mL. When detecting CTC, it is therefore of utmost importance to locate all CTC present and have an extremely sensitive and specific assay to discriminate between the tumor cells and blood cells. In the CellSearch^®^ system, epithelial cell adhesion molecule (EpCAM) antibodies coupled to ferrofluids are used for immunomagnetic enrichment of cells thereby removing the bulk of leukocytes. After this, CTC are fluorescently labeled with: 4′6-diamidino-2-phenylindole (DAPI) to stain the nucleus; phycoerythrin (PE) labeled antibodies recognizing cytokeratins (CK) 4–6, 8, 10, 13, 18, and 19, which are present in the majority of cells derived from epithelial cancers, and allophycocyanin (APC) labeled cluster of differentiation (CD) 45, which is present on the cell surface of leukocytes [10]. Images covering the surface of the CellTracks cartridge are acquired by the CellTracks Analyzer II, and all events that are positive for both DAPI and CK are presented as thumbnail images to the operator, who will decide which cells follow the criteria for CTC assignment. The identity and number of all other nucleated cells are not provided, thereby obscuring any potential cells of interest. These cells can now be explored using the open source image analysis program Automatic CTC Classification, Enumeration and PhenoTyping (ACCEPT) (download on https://github.com/LeonieZ/ACCEPT). The ACCEPT program is an open source toolbox designed to analyze and characterize all objects present in the fluorescent images.

With this program, we analyzed 659 EpCAM-enriched blood samples, obtained from 162 healthy donors or patients with benign disease and 192 metastatic non-small cell lung cancer (NSCLC) patients. We pursued several methods to determine the extent and identity of all cell populations present, in order to assess the number of cells that potentially are CTC.

We observed that the number of cells identified as leukocytes in the EpCAM-enriched fraction was low and showed a large variance across the samples. We investigated several possible causes: A possible correlation with sample or patient attributes, insufficiencies in the image analysis, and insufficiencies in the staining of the leukocytes or its detection. To improve this last option, we explored several avenues. We showed previously that addition of CD16, recognizing the FcγIIIa, to the staining cocktail improved the identification of nuclei as granulocytes since they express CD45 at a lower level compared to lymphocytes and monocytes [11]. We now applied this antibody in a large cohort of patient samples. We also explored an alternative LED excitation light source to improve the excitation efficiency of the APC fluorochrome, as opposed to the mercury arc lamp which is used as a light source in the CellTracks [12]. In addition, wheat germ agglutinin (wga), a lectin that binds to sialic acid and N-acetylglucosamine residues, was explored because it is present on cellular plasma membranes [13,14,15,16,17,18]. We examined the presence of a cell membrane by using wga conjugated to AlexaFluor-488 and adding it to the CellSearch immunostaining cocktail.

In summary, we present a method to closely examine every cell present in the cartridge after EpCAM enrichment. CTC subpopulations expressing low or no CK are currently not examined, and their potential clinical utility remains unexploited. Here we describe several methods to discover the extent of the anonymous cell population that could harbor these unidentified CTC.

## 2. Results

### 2.1. Enumeration of Nucleated Cells in the EpCAM-Enriched Cells 

**Figure 1:**
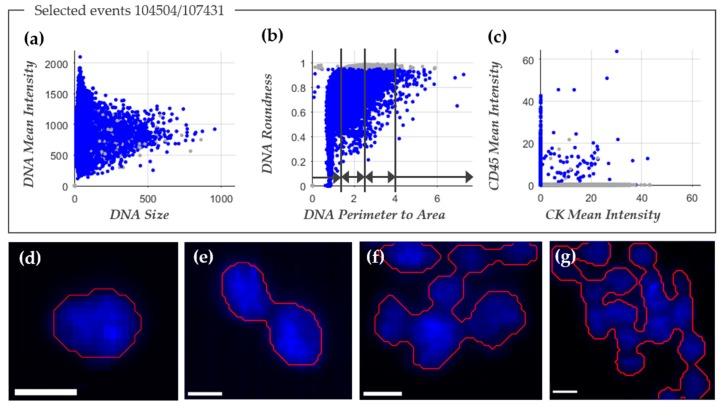
ACCEPT identification of nucleated cells. The three scatter plots in panels (**a**–**c**) were used to define nucleated cells (depicted in blue) by ACCEPT. In total, 104,504 events were detected and 107,431 of them were classified as nucleated cells, whereas the other events are depicted as grey dots. The division into single cells, doublets, small, and large clusters was based on DNA perimeter to area ratio, as illustrated in panel (**b**). In the scatter plot in panel (**c**), the mean fluorescence intensity of Cytokeratin (CK)-phycoerythrin (PE) (CK mean intensity) is plotted against the mean fluorescence intensity of CD45-allophycocyanin (APC) (CD45 mean intensity). In panels (**d**–**g**) typical examples of the segmentation (red line) around the nucleus of a single cell are illustrated in panel (**d**); of a doublet in panel (**e**); a small cluster in panel (**f**); and a large cluster in panel (**g**). The white scale bar represents 10 pixels, which corresponds to a size of 6.4 µm.

**Figure 2:**
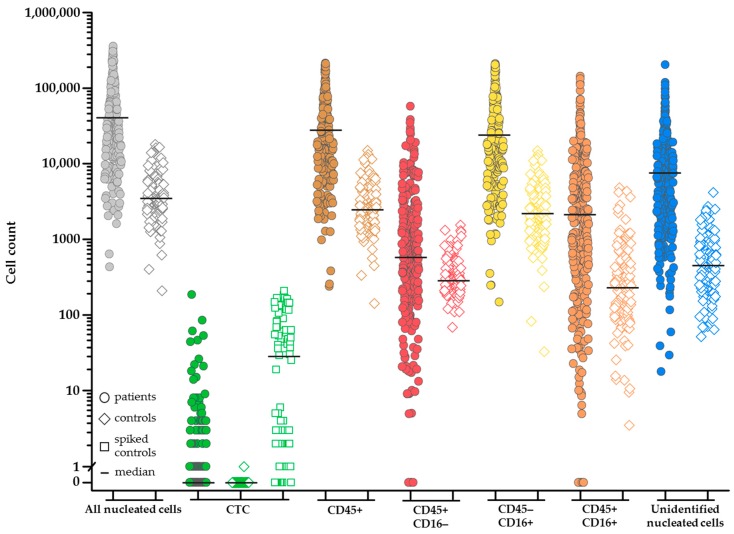
Frequency distribution of the cell populations detected in CellSearch^®^ and with the addition of the CD16 classification after epithelial cell adhesion molecule (EpCAM) immunomagnetic enrichment of 300 blood samples from 192 non-small cell lung cancer (NSCLC) patients (filled circle) and 127 blood samples from 20 healthy volunteers (open diamond). Healthy volunteer samples spiked with cell line cells (*n* = 88) are indicated with an open square. Cell populations: All nucleated cells (grey), circulating tumor cells (CTC) (green), CD45+ leukocytes (brown), CD45+/CD16- leukocytes (red), CD45-/CD16+ leukocytes (yellow), CD45+/CD16+ leukocytes (orange), and unidentified cells (blue). The differences in cell count between the NSCLC patients and healthy volunteers in the same cell classification is significant for all subclasses (*p* < 0.001). Samples from healthy volunteers were not spiked (diamond symbol, *n* = 39; median 0 CTC) or spiked (square symbol) with cancer cell lines PC3 (*n* = 47; median 63 CTC; 1.0 × 10^4^ EpCAM antigens) and NCI-H460 (*n* = 41; median 1 CTC; 1.4 × 10^2^ EpCAM antigens).

The image analysis program ACCEPT was used to enumerate the total number of nucleated cells in the image files after EpCAM enrichment. Figure 1a–c shows an example of the ACCEPT analysis in which the nucleated cells (blue dots) are identified among the other objects in the images (grey dots). To identify nucleated cells in the cartridge images, showing the following criteria for DNA staining were used: 1. Mean fluorescence intensity ≥50 (panel a); 2. standard deviation of the fluorescence intensity ≥20; 3. Size <500 µm^2^ (panel a); and 4. Roundness <0.95, where 0 is a perfect circle (panel b). To identify single cells, a perimeter to area (P2A) of <1.5 was used, for doublets a P2A between ≥1.5 and <2.5, for small clusters a P2A between ≥2.5 and <4, and for large clusters a P2A ≥4 (panel b). For enumeration of nucleated cells, the doublets and clusters were counted as one. An example of the single cells, double cells, and clusters that were identified in this manner are illustrated in Figure 1d–g. Figure 2 shows the nucleated cell count from 300 samples obtained from 192 NSCLC patients (mean 73,570 ± 74,948, median 47,192) and from 359 samples obtained from 162 healthy donors and patients with benign disease (mean 4191 ± 4463, median 2729). The number of nucleated cells was significantly larger in the patient samples as compared to the samples from healthy donors and patients with benign disease (Mann-Whitney U test: *p* < 0.001).

For a complete analysis, we determined the association between the number of nucleated cells and the sample type (“patients” or “controls”). For this analysis, 300 samples from metastatic NSCLC patients and 359 samples from healthy volunteers and patients with benign disease were used.

First, several factors were investigated that could influence the nucleated cell count since these factors were present in a limited set of patients or controls only. As the first factor, we evaluated the influence of the assay itself by determining the nucleated cell count from 30 controls with benign disease from which four blood tubes were obtained and processed simultaneously, see Figure S1. It indicated that a coefficient of variation of less than 10.7% or more than 40.2% (mean of 25.6 ± 14.9% (1 standard deviation)) of the nucleated cell count cannot be attributed to assay variation with a 97% certainty. This was only the case for six (20%) controls. The influence of the assay itself was therefore excluded from further analysis. Second, undergoing treatment as a contributing factor was considered. In 64 NSCLC patients, we observed that 95% of the patients showed an increase or decrease in nucleated events of more than 10% during treatment, compared to the nucleated events at the start of treatment, see Figure S1. We considered this a significant change and therefore this factor was included in further analysis. The third factor investigated was gender. No influence of the gender (133 male patients, 127 female patients, and 40 patients of unknown gender) on the nucleated cell count was observed with the Mann-Whitney U test (*p* = 0.237), and the factor was therefore excluded from further analysis.

In the crude multiple regression analysis for all 300 NSCLC patients and 359 controls, the relation between sample types “patient” and “control” was established. The result of this multiple regression analysis is presented in Table 1. This was followed by an analysis to correct for patient age (younger vs. older than 55 years). As a third analysis, we corrected for the patient variables: CTC count (continuous variable), treatment (during treatment vs. before or no treatment) and sample age at the time of processing with CellSearch (1 day vs. 2–4 days).

Each variable was analyzed for its confounding effect on the number of nucleated events. This showed that age of the patient, undergoing treatment, and CTC count had little confounding effect (below 5%). The sample age showed a confounding effect of 11.7%.

### 2.2. Improved Image Analysis

The number of nucleated events present in some cartridges can be enormous (over 100,000 for 27% of the patients), covering the whole detection surface, while some cartridges barely hold any cells (less than 5000 for 6% of the patients). The high number of cells creates a large area of packed cells, which makes it difficult to define borders for each cell, either manually or by image analysis algorithms. The segmentation quality of the ACCEPT toolbox was influenced by the number of cells present in the sample, an illumination bias, and fragmented staining. This is illustrated in detail in Figure S2. These problems motivated the use of a segmentation approach based on deep learning. As opposed to the model-based approaches currently implemented in the ACCEPT toolbox, deep learning approaches do not make any model assumptions about the signal (e.g., a nearly homogenous background and cell intensity), but learn a model based on only the data used for the training.

### 2.3. Assignment of Nucleated Cells to a Cell Lineage

In the CellSearch system, CD45-APC is used to identify leukocytes and Cytokeratin-PE to identify CTC among the nucleated cells. Nucleated cells expressing both CD45-APC and Cytokeratin-PE are assigned as cells binding non-specifically to at least one of the antibodies. The number of CTC identified by the classical CellSearch method in the 300 samples from 192 NSCLC patients ranged from 0–186 (mean 2; median 0) and represent only a very small portion of the nucleated cells identified in the samples from these patients. To determine whether the identity of the majority of nucleated cells could be assigned to the hematopoietic cell lineage by their expression of CD45, the thumbnails of the nucleated cells were put into a trained deep learning network for segmentation and the segmented images were fed back into the ACCEPT toolbox. Using the marker characterization processor tool, the results could be visualized and analyzed. The improved segmentation allowed for a relatively simple definition of cell subpopulations and in most cases a simple yes or no could be used to define the presence or absence of a marker. The actual definitions of the different cell populations are listed in Table 2. In this manner, nucleated cells were classified as CD45+, CD45- and CK- or CK+ in 300 samples from 192 NSCLC patients and 127 samples from 20 healthy volunteers.

**Figure 3:**
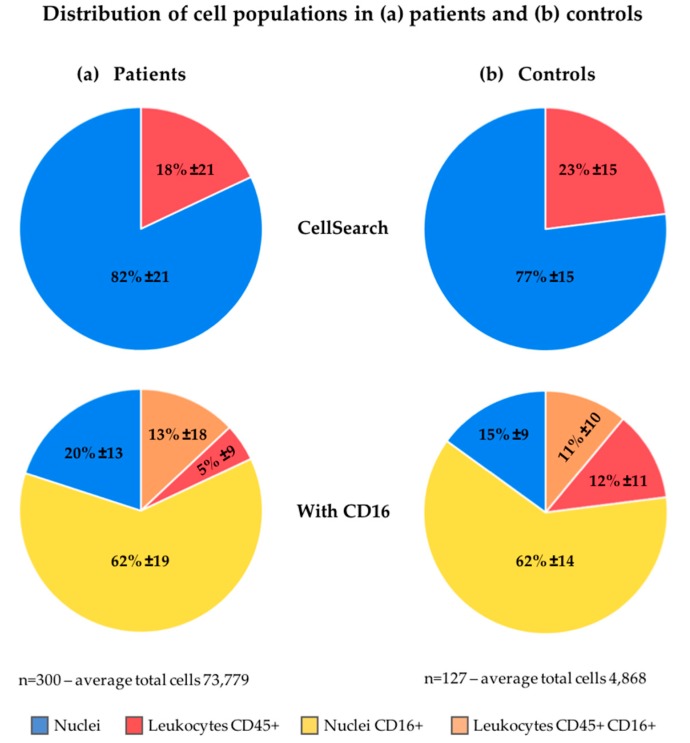
Cell population distribution in (**a**) patients and (**b**) controls using the CellSearch definition (**upper**) and using CD16 expression (**lower**) for further classification of the cells.

As described in the method section, for this analysis, 10% of the nucleated cells found by the ACCEPT toolbox, up to a maximum number of 5000 cells, were analyzed and the results were extrapolated to the full number of nucleated events to arrive at the cell numbers. The number of nucleated cells that could be identified as leukocytes in CellSearch was only 18% ± 21% (range 0–89%; median 9%) in patients and 23% ± 15% (range 2–68%; median 19%) in healthy volunteers, see Figure 3. To be able to assign the cell lineage of origin of the nucleated cells on which no CD45- or CK-expression could be detected, several approaches were pursued.

### 2.4. Increasing Leukocyte Identification by Adding CD16 Immunostaining

**Figure 4:**
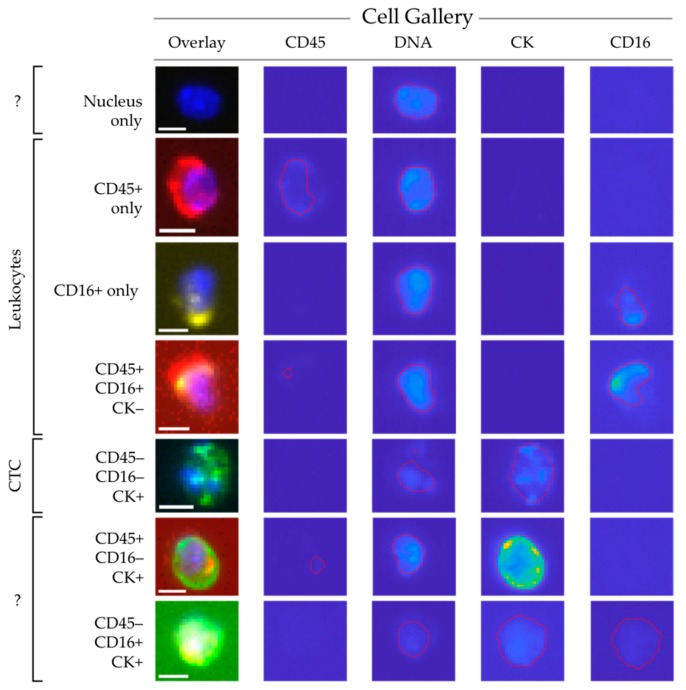
ACCEPT gallery showing cells from all classifications using the presence or absence of several markers. The scale bar is 10 pixels, representing 6.4 µm.

Whereas CD45 is basically present on all human leukocytes, it is most strongly expressed on lymphocytes and monocytes and expressed at a relatively low density in granulocytes. CD16 identifies the Fc III receptor present on granulocytes and natural killers cells. We added anti-CD16 labeled to PerCP to the CellSearch staining cocktail to improve the identification of granulocytes especially, using 300 blood samples from 192 NSCLC patients and 127 blood samples from 20 healthy volunteers. Thumbnail images of the nucleated cells identified by ACCEPT were fed into the trained deep learning network as described above. Figure 4 shows typical thumbnail images of nucleated events identified by ACCEPT using the presence of the different markers for classification. Classified CTC with expression of CD45 or CD16 can either be; (1) CTC nonspecifically binding to CD16 or CD45 antibodies; or (2) leukocytes non-specifically binding to the cytokeratin antibodies. The distribution of these cell populations in the patient and healthy volunteer samples is shown in Figure 3. From the 82% ± 21% unidentified nucleated cells in 192 NSCLC patients, 62% ± 19% are identified as granulocytes through CD16-expression. This decreases the number of unidentified cells to 20% ± 13% of the total nucleated cells present in the cartridge. The percentage of CD45-expressing leukocytes that also express CD16 is 13% ± 18%. These are most likely natural killer cells that express CD45 in higher densities compared to granulocytes. In 127 samples from 20 healthy volunteers, 77% ± 15% of the cells are unidentified which was decreased to 15% ± 9% when CD16 was used. The actual number of nucleated cells identified by either CD45 or CD16 for patients and healthy volunteers is shown in Figure 2. The number of all classifications of nucleated cells was significantly larger in patients compared to healthy volunteers (*p* < 0.001). Figure 2 and Figure 4 show there is still a large number of nucleated cells, especially in the patients, that remain unidentified.

### 2.5. LED as a Light Source to Improve Excitation Efficiency of CD45-APC and CD16-PerCP

**Figure 5:**
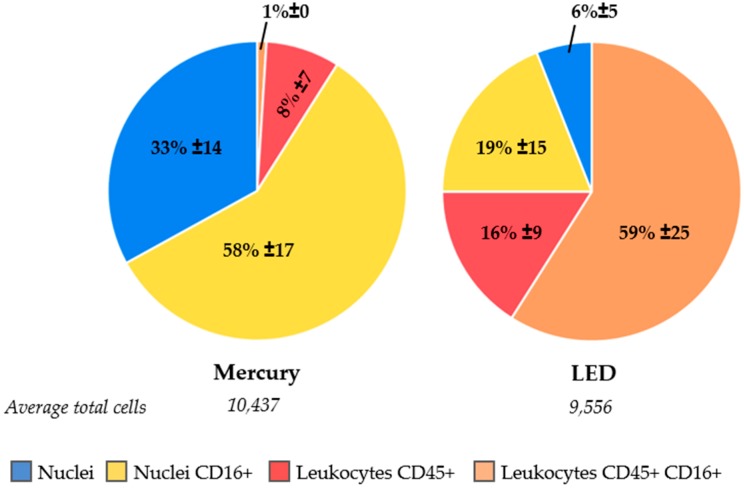
Improved identification of cells by comparison of cell population distributions analyzed with the mercury arc light source in CellSearch (**left**), followed by analysis with a LED light source on a separate microscope (**right**).

For five NSCLC patients and five healthy volunteers, cartridges were scanned first with the CellTracks–equipped with a mercury arc lamp as a light source–followed by scanning on a microscope equipped with an LED light source. The objectives and filter cubes were kept the same in both systems. The mean percentage of CD45+/CD16- leukocytes found by the CellTracks was 8% ± 7% and this increased to 16% ± 9% when a LED light source was used, as shown in Figure 5. More pronounced was the increase for CD45+/CD16+ leukocytes, which increased from 1% ± 0% to 59% ± 25% with the LED light source. Whereas CD16+/CD45- cells comprise a large population of the cells when a mercury arc lamp is used, these cells also appear to be CD45+ when the LED light source was used for the analysis; CD16+/CD45- leukocytes decreased from 58 ± 17% to 19% ± 15%. In total, the number of unidentified cells was reduced from 33% ± 14% to 6% ± 5% when an LED light source was used instead of a mercury arc lamp.

### 2.6. Identification of Unstained Nuclei by Adding Wheat Germ Agglutinin Immunostaining

**Figure 6:**
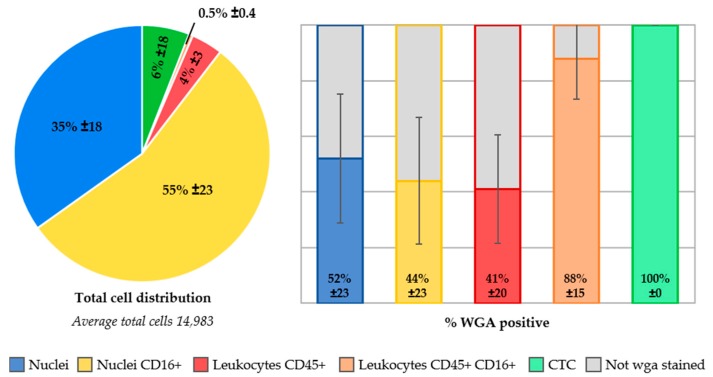
Distribution of cell populations in samples with wheat germ agglutinin (wga)-AlexaFluor488 staining (*n* = 10). The stacked bar represents the five cell populations present in these cartridges. One healthy volunteer sample was spiked with MCF-7 cancer cells, which are represented in the “CTC” category (6%). Of each population, the percentage of cells that stained positive for wga is displayed on the right side of the image, whereas the population remaining unstained with wga is visualized in grey.

In ten whole blood samples from five NSCLC patients and five healthy volunteers, the lectin wga conjugated to Alexa-Fluor488 was added to the CellSearch staining cocktail for the identification of cellular plasma membranes. In Figure S3, an ACCEPT gallery shows the staining of wga present on all cell classifications. The absence of wga on a nucleus, classifies this event as a “bare nucleus”. The presence of wga on already identified cells was 44% ± 23% for CD16+ granulocytes, 41% ± 20% for CD45+ leukocytes and 88% ± 15% for CD45+/CD16+ leukocytes, see Figure 6. One healthy volunteer sample was spiked with the cancer cell line MCF-7, and 100% of those spiked cells were stained with wga. In contrast, 52% ± 23% of the unidentified cell population was stained with wga, suggesting these are intact cells, whereas the remaining 48% ± 23% of these nuclei appear to be without a cell membrane. This suggests that these are simply bare nuclei, which cannot be identified through immunofluorescence staining.

## 3. Discussion

In this study, we investigated the cell populations present in 659 cartridges after immunomagnetic EpCAM enrichment by the CellSearch system, gathered from 162 healthy volunteers and 192 NSCLC patients. Stored fluorescent microscope images of the surface of the cartridges were analyzed by image analysis for the presence of different cell populations. The identity and number of cells present in the EpCAM-enriched cell suspension is not known, since the software only detects and presents DAPI+/CK+ events for review that are co-located. Any potential tumor cells or other interesting events remain obscured. We therefore explored the identity of all events present in the cartridge after EpCAM enrichment.

Images from the CellTracks microscope were analyzed with advanced imaging techniques. Most of the image analysis tools that were used are already assembled in the open source imaging program ACCEPT, including a tool to compare operator variability in scoring CTC [19,20]. In this image data set we had to deal with samples of low image quality, unequal distribution of the illumination, and extremely crowded images containing many clustered events, see Figure S2. A deep learning based approach appeared to be a good solution since it could easily be adapted to a certain type of signal to segment by feeding it with enough training data. The deep learning based segmentation algorithm was programmed in Python^®^ using the Keras [21] framework and is not yet linked to the ACCEPT toolbox, which is developed in MATLAB^®^. To be able to use the deep learning based segmentation, we exported thumbnails identified by ACCEPT and used them as input for the deep learning segmentation network. After segmentation of the thumbnails by the network, the segmentation results, together with the original thumbnails, were fed back into the ACCEPT toolbox for further analysis. For samples with thousands of nucleated events (up to 300,000 with five fluorescent channels each), this became quite a computationally intensive task. Therefore, for the current analysis we restricted that part of our analysis to only 10% of the nucleated events which were randomly selected. Experience obtained with the deep learning based classification algorithm we developed showed very promising results and the new segmentation method also showed that it is often superior to the more traditional segmentation approach currently used in ACCEPT [22]. Thus, in the future, we would like to incorporate a semantic deep learning segmentation into the ACCEPT toolbox to automatically detect and classify CTC, tumor derived extra cellular vesicles, leukocytes, and other cell populations for which reagents are added.

In total, 300 samples obtained from 192 metastatic lung cancer patients, 232 samples obtained from 142 patients with benign tumors, and 127 samples obtained from 20 healthy volunteers were investigated for the presence of nucleated events. The number of nucleated events was significantly higher in cancer patients as compared to the healthy donors and patients with benign disease, see Figure 2 and Figure S1, and further studies are needed to explore the potential clinical utility of this finding. CTC were detected by traditional CellSearch scoring in a small portion of the patient samples and in these samples the CTC number ranged from 1 to 186 cells. This low number of cells could not account for the large differences in the detected nucleated events.

Knowledge of the composition of these nucleated cells is needed to be able to hypothesize about this difference. Most likely, these nucleated events originate from hematopoietic cells that are captured along with the EpCAM enrichment in CellSearch. However, the fraction of nucleated cells on which the leukocyte marker CD45 was detected was remarkably low both in healthy volunteers and metastatic cancer patients, as shown in Figure 3. Since the excitation of CD45-APC with a mercury arc lamp used in the CellTracks system is not optimal, we first looked into a way to increase the detection of granulocytes especially, which express the CD45 antigen in relatively low antigen density compared to leukocytes.

We evaluated this by adding anti-CD16-PerCP antibody to the immunostaining in the CellSearch system. All 300 samples from metastatic lung cancer patients and 127 samples from healthy volunteers were processed with this extra marker. The leukocytes and granulocytes that were identified indeed increased, as shown in Figure 3. However, a considerable number of nucleated cells remained unidentified. Next, we looked into the role of ferrofluids, which are used at a concentration of 40 µg/mL in CellSearch and are present on the imaging surface of the cartridge [23,24]. The ferrofluids reduce the intensity of the fluorescence; an effect which is most obvious in the blue region (400–460 nm). Therefore, we looked into an improvement of the excitation of the fluorophores. For this, a microscope equipped with an LED light source to improve the APC fluorophore excitation was used [12]. This resulted in a clear decrease of unidentified nucleated cells, see Figure 5. Also, to determine whether these remaining nuclei were enclosed by a cellular plasma membrane, we added wheat germ agglutinin to the CellSearch cocktail [13,14]. This showed that part of the unidentified nucleated cells did not stain with wga, suggesting they are “bare”-like nuclei, see Figure 6.

Whether or not these bare nuclei were actually present in the blood, or if they represent cells that have been damaged through the immunomagnetic selection or staining process, remains an outstanding question. However, a large fraction of leukocytes identified with the current immunostaining are not also stained with wga. This could suggest that the wga-staining assay is not sufficiently sensitive to stain all membranes or that the imaging method is not sufficiently sensitive to detect dim wga fluorescence. A high concentration of wga added to the CellSearch cocktail caused the aggregation of blood cells, and therefore a lower concentration was chosen for this assay. It might, however, be possible that a higher concentration of wga is necessary to locate all membranes present in the blood sample. Also, all samples were analyzed on the CellTracks, but improved identification will be observed when the LED light source is used. Another option is that the leukocyte membranes are damaged to a point that the agglutinin can no longer bind to the residues on the membrane. This may be caused during the 15 min permeabilization process to open the membranes. The CellSearch permeabilization agent is saponin, a detergent which interacts with membrane cholesterols by selectively removing them to form pores, which enables antibodies to enter [25]. It is notable, that the fraction of the spiked MCF-7 cancer cells that were stained with wga was 100%. Compared to cancer cell line cells, the leukocytes are possibly more fragile and susceptible to damage caused during permeabilization.

The wga+/DNA+/CD45-/CD16-/CK- cells are potentially CTC that are not expressing CK for CellSearch detection. However, our observation that a similar population was detected in the blood of healthy volunteers makes this explanation less likely. Confirmation of whether or not CTC are among the unidentified nucleated cells will likely have to come from DNA or RNA assays. Removing the cells from the cartridge might allow them to be selected based on their absence of fluorescence markers and subsequently sorted with fluorescence-activated cell sorting (FACS). Quantitative PCR after DNA isolation and amplification could reveal the presence of mutations, indicating one of the cells is, in fact, a tumor cell. Digital-droplet PCR has a higher sensitivity and could be applied on the entire cell suspension from a cartridge, although the multiplex analysis of mutations is less feasible in this assay. With these approaches, the number of tumor cells will have to be compared to the number of CTC scored on the CellTracks (if present at all) to determine if more or an equal number of tumor cells are present in the cell suspension. Also, analysis of mRNA after FACS isolation might be feasible for the unidentified cells with a cellular membrane present. Quantification of a selection of proteins can indicate if the cells are of hematopoietic of epithelial lineage. However, the permeabilization of cells in the CellSearch might complicate this approach. Therefore, the CellSearch Epithelial Cell Profile Kit should be used. This kit is designed for RNA analysis and enriches EpCAM+ cells without subsequent permeabilization and staining.

After taking several steps to determine the origin of unidentified cells, the possible identity for the cells that remained unidentified thus far might be:

1. Hematopoietic cells not identified through damage incurred during the procedure or inaccessible antigens through the ferrofluids [23,24];
2. Hematopoietic cells without sufficient expression of CD45 and CD16: This suggests they would need additional CD markers for identification or an improved labeling method that amplifies low signals, which might yield increased detection of very dim stained cells and separate the fluorescent signal of densely packed cells [11,26,27,28]. Early myeloid cells have recently been observed to surround the tumor in high numbers, and as these cells do not yet express CD16, this remains a possibility [29];
3. CTC with no or low expression of the CK antigens detected by the C11 and A53.B/A2 clones: Since these clones only recognize a subset of the CK present in a cell, it might be beneficial to include antibody clones that recognize all CK. Previously, we have shown that adding several CK clones to the CellSearch antibody cocktail improved the detection of CTC positive patients by 11% [30]. Also, it might be possible that EpCAM+/CK- CTC are present, remaining undetected because of the downregulation of epithelial markers through epithelial-to-mesenchymal transition [31,32,33]. In order to detect these cells, antibodies specific to this process could be added to the CellSearch immunostaining [34,35,36];
4. Cells of other origin: Such as circulating stromal, endothelial, or stem cells [37]. Detection of these cells would also require the addition of other antibodies to the assay.

Therefore, there is still much to be achieved toward the identification of the cells that remain anonymous. It is most important to determine if all tumor cells have been located or if some are still missed.

## 4. Materials and Methods

### 4.1. Cancer Patients, Patients with Benign Disease, and Healthy Volunteers

Peripheral blood samples were drawn by venipuncture into 10 mL CellSave Preservative Tubes (Menarini Silicon Biosystems, Huntingdon Valley PA, USA) from healthy volunteers and metastatic NSCLC patients. A total of 300 blood samples from 192 NSCLC patients were obtained after patients with advanced NSCLC provided written informed consent. The medical ethical committee of the University Medical Center Groningen and Board of Directors approved the protocol (project number 288.194/7 April 2014; Groningen, The Netherlands). Blood draw from patients occurred before and/or during their treatment. A total of 127 blood samples from 20 healthy volunteers aged 20–55 were obtained from the donor services of the Experimental Centre for Technical Medicine at the University of Twente, and all donors gave written informed consent before donating blood. All healthy volunteer samples were processed 1 day after blood draw, whereas patient samples were processed after 1–4 days. Digitally stored images from 232 samples from 142 patients with benign disease processed with CellSearch from previous studies were included for ACCEPT analysis [10,38].

### 4.2. Cell Lines and Spiking

Whole blood samples from healthy volunteers were spiked with cancer cell lines PC3, NCI-H460, and MCF-7. PC3 (1.0 × 10^4^ EpCAM antigens) and NCI-H460 (1.4 × 10^2^ EpCAM antigens) were used for frequency detection of CTC with ACCEPT and MCF-7 (8.8 × 10^5^ EpCAM antigens) was used for testing wheat germ agglutinin (wga) staining of cancer cells. This cell line was chosen for its high EpCAM expression to ensure sufficient capture of cancer cells in the cartridge for analysis. All cell lines were obtained from ATCC (Manassas, VA, USA) and have not been authenticated in the past six months. The cells were grown at 37 °C and 5% CO_2_ and cultured in RPMI-1640 (Sigma-Aldrich, St. Louis, MO, USA). The culture media was supplemented with L-Glutamine (Sigma), 10% fetal bovine serum (Gibco, Invitrogen, Carlsbad, CA, USA), and 1% penicillin-streptomycin (Gibco, Invitrogen, Carlsbad, CA, USA).

### 4.3. Processing Blood with CellSearch

Aliquots of 7.5 mL of blood were processed with the CellSearch^®^ system (Menarini Silicon Biosystems, Castel Maggiore (BO), Italy) for CTC detection. CellSearch analysis was performed within 96 h after blood was drawn. Antibodies directed against the epithelial cell adhesion antigen (EpCAM) coupled to ferrofluids were used to enrich CTC from the background of leukocytes. The enriched cells were fluorescently labeled with the CellSearch CTC kit (Menarini Silicon Biosystems) using the nucleic acid dye 4′6-diaminodino-2-phenylindole (DAPI) for DNA staining, anti-cytokeratin monoclonal antibodies (mAbs) C11 and A53.B/A2 labelled with phycoerythrin (PE), and anti-CD45 mAb (clone HI30) labeled with allophycocyanin (APC) for recognizing leukocytes. Peridinin Chlorofyll A Protein (PerCP) labeled mAb anti-CD16 (clone 3G8, Biolegend, San Diego, CA, USA) directed against granulocytes, and Alexa-Fluor488 conjugated to the lectin wga (Thermo Fisher Scientific, Waltham, MA, USA) were added to the extra marker position in the CellSearch Epithelial Cell kit with an end concentration of 2 µg/mL and 3 µg/mL, respectively. This extra marker channel was used for the measurement of PerCP and DIOC on the CellTracks Analyzer II, which generates images of the complete cartridge for all channels.

### 4.4. Image Acquisition

Fluorescent microscopy images were acquired using a modified CellTracks Analyzer II, which included a 20× and 40× objective and in total six filter cubes next to the standard instruments. Besides the acquisition of the standard channels DAPI, PE, DIOC, and APC, the images with PerCP staining were acquired with a customized PerCP filter cube (excitation 435/40 nm; dichroic 510 nm; emission 676/29 nm (Semrock, Rochester, NY, USA)) using an integration time of 400 ms. When wga was used in the immunostaining, the integration time of the DIOC filter was also 400 ms. To determine if another light source detects weakly stained leukocytes better than the mercury arc lamp of the CellTracks, ten cartridges were automatically scanned with an inverted Nikon Ti-E microscope with filter cube and objective changer (Nikon, Tokyo, Japan). The light source of this microscope is the Lumencor Sola SE II 365 LED. A 10× (0.45NA) objective (Nikon, Tokyo, Japan), a computer-controlled CCD camera (C114400, Orca Flash 4.0LT, Hamamatsu Photonics, Hamamatsu, Japan), an ASI MS-2500 XY stage and filters optimized for LED illumination were used for the acquisition of fluorescent images of the cartridge. The following filters (all from Semrock) were used: DAPI with excitation 377/50 nm, dichroic 409 nm, emission 409 nm/LP; fluorescein isothiocyanate (FITC) with excitation 482/18 nm, dichroic 495 nm, emission 520/28 nm; PE with excitation 543/22 nm, dichroic 562 nm, emission 593/40 nm; APC with excitation 635/18 nm, dichroic 652 nm, emission 680/42 nm; and PerCP with excitation 435/40 nm, dichroic 510 nm, emission 676/29 nm. The integration times were the same as used in the CellTracks: For DAPI 30 ms, FITC 100 ms, PE 200 ms, APC 600 ms, and for PerCP 400 ms. The scanning and image acquisition was controlled by a software program written in Labview (National Instruments, Austin, TX, USA).

### 4.5. Image Analysis with ACCEPT

In CellSearch, CTC are annotated when the objects are stained with DAPI and CK, but lack CD45 staining and are larger than 4 µm with morphological features consistent with those of a cell [10,38]. In the ACCEPT (Automated CTC Classification, Enumeration, and PhenoTyping) toolbox, every event in the images is analyzed using an advanced multi-scale segmentation approach and several intensity and shape measurements are extracted for every event found [19,39]. ACCEPT is developed in the EU funded “CANCER-ID” and “CTC-Trap” programs, as an open source toolbox for CTC analysis and can be downloaded from https://github.com/LeonieZ/ACCEPT. New tools and features for ACCEPT are being developed and made available in the downloadable version at regular intervals.

For this study, the fluorescent images from all samples were uploaded, and all events present in the images were detected. To enhance the detection of cells, especially in areas where the cells formed large cell clusters, we added a background subtraction to the ACCEPT toolbox. Before images are segmented, a Fourier filtering is applied, and large and very fine scales are filtered by multiplying the filtered signal with two Gaussian kernels before the signal is back-transformed by an inverse Fourier transformation. In this way, the background is removed, which enhances the detection in very crowded areas, as well as areas with inhomogeneous illumination.

Fragmented fluorescent signals make a segmentation with an intensity-based segmentation method difficult, which is now implemented in the downloadable version of the ACCEPT toolbox. In this study this was especially observed in the fluorescent images obtained from the CD16-PerCP stained cells. Therefore, we decided to explore deep learning approaches to determine if this limitation could be overcome. For this approach, all thumbnails containing nucleated cells in the cartridge were found using ACCEPT, and a random selection of these thumbnails was used for further evaluation. Per sample, we exported 10% of the observed cells, but minimally 1000 (if the total amount was high enough) and maximally 5000 thumbnails. Moreover, we exported 759 thumbnails with five fluorescent channels each where the segmentation method of ACCEPT gave satisfactory results. We used these thumbnails (split into single channels) to train a deep learning network to segment the cells. The implementation was done in Keras and we used the U-net architecture with skip connections [21,40]. The set of 3796 thumbnail images was split into a training set of 3037 images (80%) and a validation set of 759 images (20%). The images were preprocessed in the following way: Each thumbnail exported from the ACCEPT toolbox was transformed into a thumbnail of 80 × 80 pixels by adding a padding at all four sides with the median intensity of the pixels at the image boundary. Moreover, all images scanned with CellTracks were cast from double to uint8 arrays by first dividing by 4095 (the maximum value in Celltracks) and then multiplying by 255 and converting the results to uint8. Images scanned with the inverted Nikon Ti-E microscope were recorded as uint16, thus having a theoretical maximum value of 65,535. However, during our analysis, we noticed that this range was not used and that the range of intensity values used strongly differs between the fluorescent channels. Therefore, we calculated for each fluorescent channel the intensity value that was higher than 99.9% of the measured intensities over all images in all samples. The images were then divided by the resulting intensity per channel, multiplied by 255 and cast to uint8. By taking the value that is above 99.9% instead of the maximum, we accounted for individual saturated pixels where the incident light caused the camera sensor to respond with the highest possible value. To train the deep learning network, we used a batch size of 16 and the network was trained for ten epochs. The resulting convolutional network was then used to segment the exported thumbnails for each sample. The original thumbnails together with the segmentation derived by deep learning were fed back into the ACCEPT algorithm by using the marker characterization processor for further analysis and visualization of results.

### 4.6. Statistical Analysis

Statistical analysis was performed using SPSS (version 24, SPPS Inc., Chicago, IL, USA). A *p*-value below 0.05 was considered significant. The non-parametric Mann-Whitney U test with significance level α = 0.05 was used to determine if there was a difference in nucleated cell numbers between cohorts of patients and controls. The total nucleated cell amounts were log-transformed for normality. With multiple regression analysis, we determined the association of several variables with the total nucleated cell count, such as age, treatment, CTC score, and sample age before processing. The confounding effect of each variable was also determined.

## 5. Conclusions

Advanced image analysis was used to identify the cell populations present after immunomagnetic EpCAM enrichment by the CellSearch system from 659 cartridges from NSCLC patients. A significantly larger number of nucleated cells were found, from which the origin could not be identified, compared to samples from healthy volunteers and patients with benign disease. A large portion of the unidentified nucleated cells could be identified by: Adding CD16 to improve the identification of granulocytes; the use of an LED light source to improve fluorescence detection and; the addition of wga to discriminate between bare nuclei and cells. However, there is still much to gain in the identification of the cells that still remain anonymous. These observations must also be confirmed in patients with other cancers. The question remains: What are these cells? Going forward, it is most important to determine if all tumor cells have been located or if some are still missed and reside in this unidentified cell population. This unidentified CTC subpopulation could be of interesting clinical value and remains thus far unexploited. Therefore, further studies are needed to determine if EpCAM+/CK- CTC are present among the nucleated cells of which the origin is not known.

## Figures and Tables

**Table 1:** Multiple regression analysis for association between sample type and nucleated events corrected for several patient variables.

Analysis Type	Regression Coefficient	95% Confidence Interval	*p*-Value
Crude analysis	17.0	14.1–20.6	<0.001
Corrected analysis for *age*	19.2	15.3–24.2	<0.001
Corrected analysis for *age*, and patient variables (*sample age*, *CTC count* and *treatment*)	12.4	9.4–16.4	<0.001

**Table 2:** The definition of cell populations used to classify cells in ACCEPT after deep learning segmentation in 300 non-small cell lung cancer (NSCLC) patient samples and 127 samples from healthy volunteers.

Cell Population		Mean Intensity Value					Overlay Nucleus			Size (µm^2^)
		*DAPI*	*CD45*	*CK*	*CD16 ^1^*	*wga ^1^*	*with CK*	*with CD45*	*with CD16*	*CK*
**CTC**	CK+/CD45-	>0	0	≥50	0	>0	>0.4	ND	ND	>9
**Leukocytes**	CD45+/CD16-	>0	>0	0	0	>0	ND	>0	ND	ND
	CD45+/CD16+	>0	>0	0	>0	>0	ND	>0	>0	ND
	CD45-/CD16+	>0	0	0	>0	>0	ND	ND	>0	ND
**Nucleus**	Bare nucleus	>0	0	0	0	0	ND	ND	ND	ND
	Unstained cell	>0	0	0	0	>0	ND	ND	ND	ND

*^1^* Only for those samples in which CD16 or wga was added to the immunostaining cocktail. Abbreviations used: DAPI: 4′6-diamidino-2-phenylindole; CD: cluster of differentiation; CK: cytokeratins; wga: wheat germ agglutinin.

## Supplementary Materials

The following are available online at http://www.mdpi.com/2072-6694/10/10/377/s1, Figure S1: Nucleated cell count in controls with benign disease and NSCLC patients, Figure S2: Illustration of the issues that influence the segmentation quality of the ACCEPT toolbox, Figure S3: ACCEPT gallery of cells stained with the membrane lectin wheat germ agglutinin (wga).
