# Supplementary material for: Classification of Cells in CTC-Enriched Samples by Advanced Image Analysis
Source: Cancers (Basel). 2018 Oct 10;10(10):377. doi: 10.3390/cancers10100377 (PMC6210778; doi:10.3390/cancers10100377)
Supplement: Supplementary file 1 [file cancers-10-00377-s001.pdf]

Supplementary Materials: Classification of Cells in CTC Enriched Samples by Advanced Image Analysis

Sanne de Wit, Leonie L. Zeune, T. Jeroen N. Hiltermann, Harry J.M. Groen, Guus van Dalum and **Leon W.M.M. Terstappen**

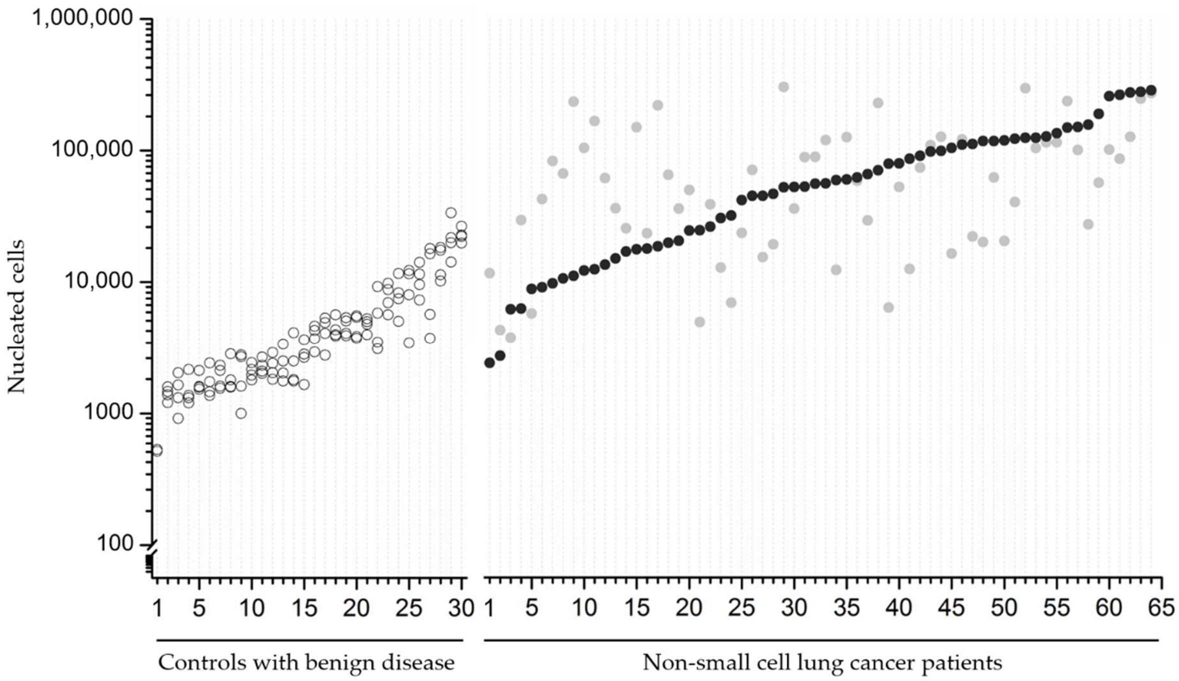

Figure S1. Nucleated cell count in patients with benign disease and non-small cell lung cancer (NSCLC) patients. Counts for four blood samples drawn at the same time in 30 patients with benign disease (a, open circle) and in 64 NSCLC patients with two blood samples (**b**): one drawn before initiation of therapy (right, dark circle) and one during therapy (light circle). For (**a**), the nucleated cell count in these samples was on average 5541 ± 541 and a median 3237; the coefficient of variation ranged from 9.9%–77.6% (mean 25.6% ±14.6%). In (**b**), 46% of the 64 NSCLC patients showed an increase of more than 10% in nucleated cell count, in 31 (48%) a decrease of more than 10% and in 3 (5%) patients the change in the nucleated cell count was not more than 10%.

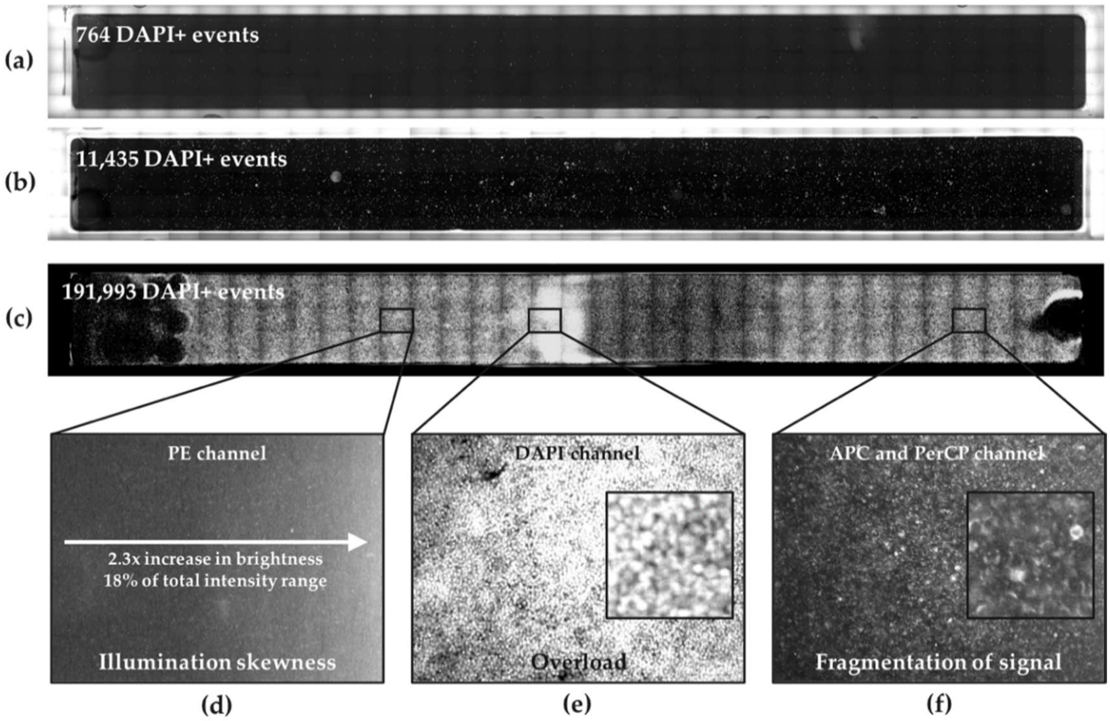

Figure S2. Illustration of the issues that influence the segmentation quality of the ACCEPT toolbox that were improved with a Deep Learning segmentation approach. In total, 175 images of DAPI staining cover the surface of CellSearch cartridges for each sample. In (**a**) a cartridge containing less than 1000 DAPI+ events are shown; (**b**) shows a cartridge containing around 10,000 DAPI+ events and; (**c**) shows a cartridge containing much more than 100,000 DAPI+ events. While single cells can easily be detected in panels (**a**) and (**b**), the example in panel (**c**) demonstrates the effect that a high cell density makes the separation of cell boundaries nearly impossible. In (**d**–**f**) three of the 175 images of the very full cartridge are shown at a higher magnification to illustrate the three main difficulties for an image analysis program to identify objects. In (**d**) the illumination skewness of the PE channel corresponding to the DAPI image in (**c**) is presented. The background intensity, averaged over a small square of 5 by 5 pixels, is 2.3 times brighter on the right side of the image compared to the left one. In relation to the overall intensity range of 4095 intensity values, this is an increase of 18%. The segmentation model used assumes a nearly homogeneous background, but this assumption fails in case of an illumination bias. Panel (**e**) shows an overload of signal because the cartridge is too full with cells to detect cellular boundaries. Panel (**f**) shows the fragmentation of signal, which makes it for the software hard to accurately segment a cell and extract correct parameters, especially in combination with very packed areas as shown in panel (**e**). The segmentation results in case of overload (**e**) or fragmentation of signal (**f**) can be improved with a Deep Learning segmentation approach.

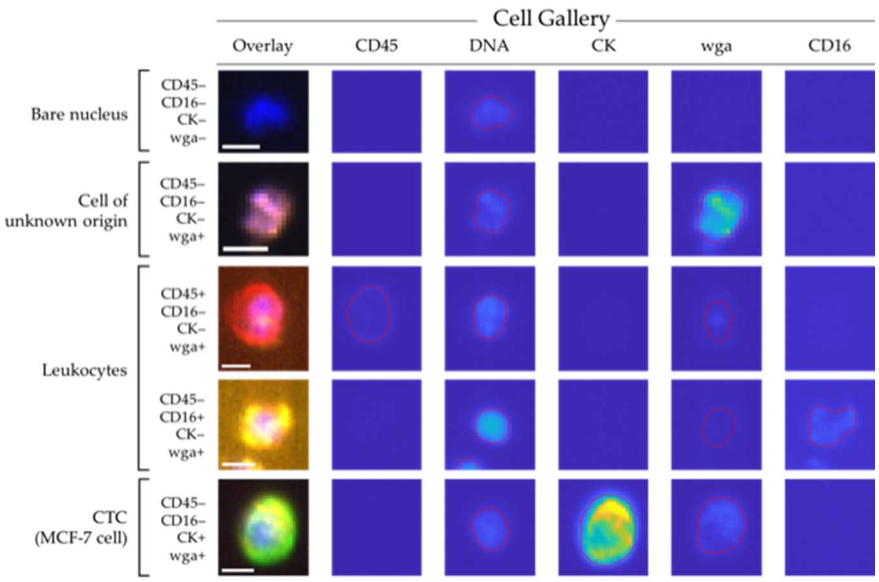

Figure S3. ACCEPT gallery of cells stained with the membrane lectin wheat germ agglutinin (wga). The cell with wga-staining, but without the presence of any other markers, remains without determined origin, whereas the nucleus without wga-staining appears to be a bare nucleus. The scale bar is 10 pixels, representing 6.4 µm.
